# Most people do not ignore salient invalid cues in memory-based decisions

**DOI:** 10.3758/s13423-012-0248-4

**Published:** 2012-05-04

**Authors:** Christine Platzer, Arndt Bröder

**Affiliations:** Experimental Psychology, University of Mannheim, Schloss EO, 68131 Mannheim, Germany

**Keywords:** Decision making, Memory, Format effects, Salience

## Abstract

Former experimental studies have shown that decisions from memory tend to rely only on a few cues, following simple noncompensatory heuristics like “take the best.” However, it has also repeatedly been demonstrated that a pictorial, as opposed to a verbal, representation of cue information fosters the inclusion of more cues in compensatory strategies, suggesting a facilitated retrieval of cue patterns. These studies did not properly control for visual salience of cues, however. In the experiment reported here, the cue salience hierarchy established in a pilot study was either congruent or incongruent with the validity order of the cues. Only the latter condition increased compensatory decision making, suggesting that the apparent representational format effect is, rather, a salience effect: Participants automatically retrieve and incorporate salient cues irrespective of their validity. Results are discussed with respect to reaction time data.

**Electronic supplementary material:**

The online version of this article (doi:10.3758/s13423-012-0248-4) contains supplementary material, which is available to authorized users.

Most decision objects are characterized by multiple pieces of information, often called *cues* (e.g., the fat and sugar content of different food items). Sometimes, there is conflicting information, and judgment and decision-making researchers have described many cognitive strategies for solving such conflicts and reaching a decision (Gigerenzer & Todd, 1999; Payne, Bettman, & Johnson, 1993). Compensatory strategies such as the “weighted additive rule” (WADD) and the “equal weight rule” (EQW) face this conflict and weigh cue information against each other, whereas noncompensatory strategies avoid this conflict by relying on a subset of information. For example, the “take the best” strategy (TTB; Gigerenzer & Todd, 1999) examines only the most valid cue, which is the best predictor of the criterion in a first step. If the cue discriminates between objects in a decision task, no further cue information is considered, and decisions are based on this cue. If this cue does not discriminate, the next most valid cue is considered, and so on.

To examine the strategies people use in actual decisions, researchers have relied on experiments in which cue information is provided to participants (e.g., MouseLab paradigm; Payne, Bettman, & Johnson, 1988). However, Gigerenzer and Todd (1999) pointed out that this research has neglected the fact that decisions often have to be made from memory. They supposed that costly retrieval of information from memory triggers noncompensatory decision making. Interestingly, only a few studies have examined decision strategies with memory-based retrieval (Bröder & Gaissmaier, 2007; Bröder, Newell, & Platzer, 2010; Bröder & Schiffer, 2003, 2006; Glöckner & Hodges, 2011; Khader et al., 2011; Persson & Rieskamp, 2009).

Bröder and Schiffer (2003) corroborated the hypothesis of more noncompensatory decision making by showing that participants were more likely to use TTB in a memory retrieval condition than in conditions in which cue information was provided on screen. However, this effect was found only when cue information was presented in a verbal cue format. Providing participants with pictorial cue information in a memory-based task resulted in more compensatory decision making. Bröder and Schiffer (2006) discussed two possible explanations for what they referred to as the *format effect*. One explanation builds on the assumption that pictorial information can be retrieved faster and more easily because it is stored in a holistic internal code (Paivio, 1978; Seifert, 1997). Since holistically stored knowledge can be retrieved in parallel, retrieval costs are low, which should allow for a more complex, compensatory integration of cue knowledge. This explanation, however, has not been corroborated by the response time data indicating no difference between a verbal and a pictorial condition, which should have been the case if the *picture superiority* explanation is correct. A second explanation is based on the assumption that both verbal and pictorial information are retrieved sequentially; thus, no reaction time differences are expected. According to this explanation, pictures convey semantically richer information, as compared with simple verbal labels (e.g., “That shirt doesn’t suit her”). Retrieval costs might therefore be reduced, thus enabling compensatory cue integration. Hence, verbal, as well as pictorial, information may be retrieved sequentially, with pictorial information being easier to retrieve as a result of a more comprehensive knowledge representation.

Both explanations are based on the assumption that decision times are exclusively determined by retrieval costs. However, it is difficult to distinguish between the two explanations on the basis of decision times. Since compensatory strategies integrate more information than does TTB, reaction times should be higher due to information integration, hence leveling out potential reaction time differences between a verbal and a pictorial condition.

In this article, we provide an alternative explanation for the results reported by Bröder and Schiffer (2003, 2006) by showing that cue validity and cue salience were confounded in their experiments. In a pilot study, we measured the visual salience of the pictorial cues used by Bröder and Schiffer (2003, 2006). The results indicated that a less valid cue was more salient than the most valid cue.^1^ Hence, retrieval costs for less valid cues might have been reduced not because they were presented pictorially, but because they were particularly salient. If the format effect was, rather, a salience effect, facilitated retrieval of pictorial cues would depend neither on the internal code nor on the semantic richness of the knowledge representation, but merely on cue salience. Since less valid but salient cues come to mind more easily, compensatory decision making should be facilitated. Additionally, decision making could be impaired if to-be-ignored invalid cues are particularly salient. For example, nervous behavior in a police interview is a salient cue for potential lying, but its validity is close to zero (Vrij, Granhag, & Porter, 2010). The assumption that irrelevant knowledge is activated even when TTB is used was corroborated by recent neuroscientific findings (Khader et al., 2011). Participants were trained to rely exclusively on the most valid discriminating cue, but brain areas associated with irrelevant cue information also showed above-control activation when participants made memory-based decisions in the fMRI scanner.

The aim of the present experiment was to disentangle format and salience effects in memory-based decisions by controlling for visual salience more thoroughly. Cue validity and salience were either positively or negatively correlated. If more compensatory decision making were to be observed in both pictorial conditions, the format effect would be properly named. If, however, more compensatory decision making were a result only in a pictorial condition in which cue validity and salience were negatively correlated, the salience hypothesis would be confirmed. In this case, reaction time data can be used to validate the salience hypothesis. In general, TTB should be faster than compensatory strategies, since less information has to be considered. If, however, validity and salience are negatively correlated, decision times for TTB should increase, because cognitive effort is needed to inhibit salient information that is irrelevant for a noncompensatory strategy. On the other hand, we would expect shorter reaction times for compensatory strategies, since less valid cues might be retrieved faster if they are particularly salient. Hence, we would expect an interaction between experimental condition and decision strategy concerning decision times.

## Experiment: Disentangling validity and salience

In the experiment, the validity of the format effect was tested against the assumption of a mere salience effect.

### Method

#### Participants

Sixty-seven students from the University of Mannheim participated in the experiment (48 of them female; mean age, 21.37 years, *SD* = 2.32). Participants received course credit, and the ten participants with the best memory performance additionally received 25 Euros each.

#### Materials and design

We used the same cover story as Bröder and Schiffer (2003, 2006) of an invented criminal case. A famous singer had been killed by one of his former girlfriends, and participants were invited to help a police officer to find the alleged murderer. Ten suspects were presented, and participants were required to decide about the probability that they had committed the crime by using information about clothes as cues. The female suspects differed with regard to the articles of clothing they had worn at the time the actual perpetrator left the site of crime. Different types of coats (leather jacket, cardigan, blazer), tops (polo shirt, shirt, blouse), trousers (jeans, leggings, linen trousers), and bags (tote bag, hand bag, wrist purse) served as cues. Table 1 shows the distribution of cue values among the ten suspects. Cue patterns were chosen such that the decision strategies TTB, WADD, and EQW made different predictions in the decision phase and, therefore, allowed for strategy classification.

**Table 1:** Cue patterns used in the experiment (adopted from Bröder & Schiffer, 2003, 2006)

Cue patterns	Cue 1	Cue 2	Cue 3	Cue 4
Pattern 1	1	1	0	0
Pattern 2	1	0	1	1
Pattern 3	1	0	1	0
Pattern 4	1	0	0	1
Pattern 5	1	0	0	0
Pattern 6	0	1	1	1
Pattern 7	0	1	1	0
Pattern 8	0	1	0	1
Pattern 9	0	0	1	1
Pattern 10	0	0	1	0

*Note*. 1 denotes a critical cue value, and 0 denotes a noncritical cue value

We conducted a pilot norming study to determine the visual salience of the four cues used in the experiment. The study entailed similarity ratings between suspects that were regressed on cue differences. Visual salience was operationalized as the extent to which the mismatch on a certain cue affected similarity ratings. The pilot study is fully documented in the supplementary materials. Regression weights indicated a distinct salience hierarchy, with ”coat” being the most salient cue, followed by ”top,” ”trousers,” and ”bag.”

**Fig. 1:**
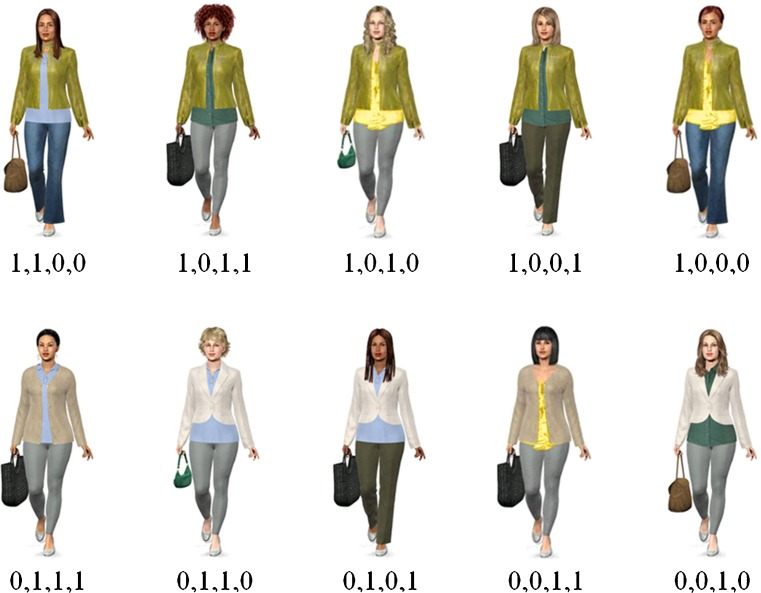
Stimuli used in the pictorial condition with a positive correlation between salience and validity. Values beneath the pictures indicate cue patterns, with 1 denoting a critical cue value and 0 denoting a noncritical cue value. Critical cue values were (in descending order of validity) leather jacket, polo shirt, leggings, and tote bag

**Fig. 2:**
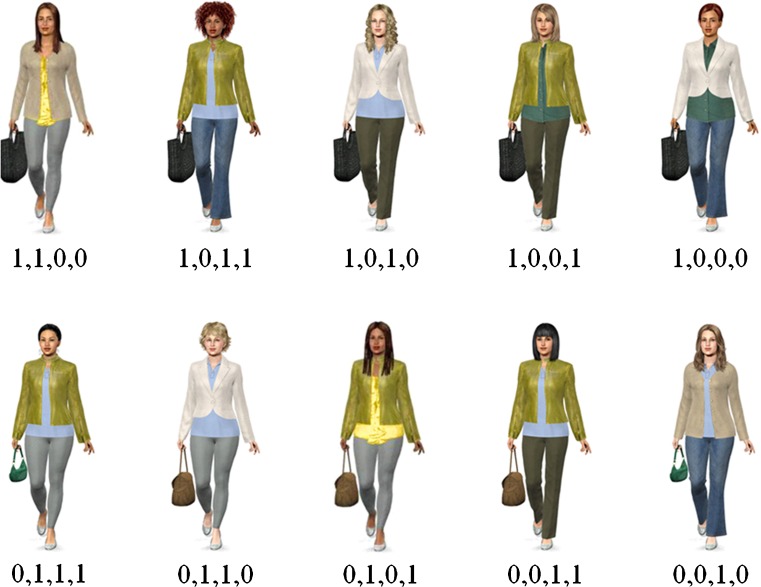
Stimuli used in the pictorial condition with a negative correlation between salience and validity. Values beneath the pictures indicate cue patterns, with 1 denoting a critical cue value and 0 denoting a noncritical cue value. Critical cue values were (in descending order of validity) tote bag, leggings, polo shirt, and leather jacket

A 2 (cue format: verbal vs. pictorial) × 2 (congruency of validity and salience: congruent vs. incongruent) between-subjects design was used, resulting in four experimental conditions: congruent verbal (CV), incongruent verbal (IV), congruent pictorial (CP), and incongruent pictorial (IP). In conditions with a verbal cue format, information about cue values was given verbally for each suspect. In conditions with a pictorial cue format, pictures of suspects were shown. Figures 1 and 2 illustrate the stimuli used in both pictorial conditions. In the CP condition, validities matched the salience ratings, resulting in the following validity hierarchy of cues: coat, top, trousers, and bag. The validity hierarchy in the IP condition was inverted, with bag being the most valid (but least salient) cue, followed by trousers, top, and coat. Note that the stimuli differed in both pictorial conditions as a consequence of different validity hierarchies.^2^ This potential confounding of salience and semantic content was controlled for by also using two verbal conditions. Cue values were presented as verbal descriptions of the pictorial cue values used in the CP condition (CV) and IP condition (IV). Hence, different stimuli were used in the CV and IV conditions to control for the influence of stimulus materials. Note that we did not manipulate the congruency of validity and salience in the two verbal conditions, since verbal descriptions do not differ in visual salience.

#### Procedure

**Fig. 3:**
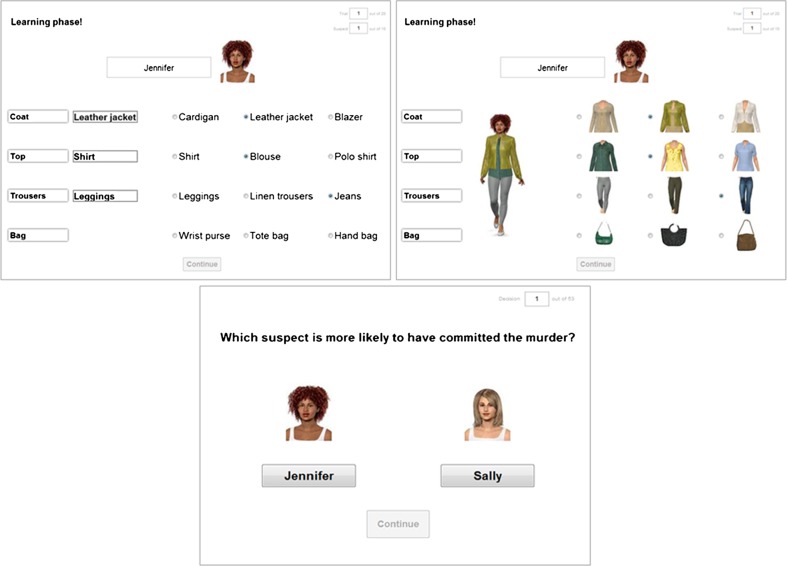
First row: Sample trial of the learning phase in the verbal condition (left screenshot) and in the pictorial condition (right screenshot). Both conditions show a trial with positive correlation between validity and salience (CV, CP). Second row: Sample trial of the decision phase, which was identical in all conditions

The procedure was identical to that in Experiment 3 in Bröder and Schiffer (2003). In a learning phase, participants learned cue patterns of the ten suspects in an anticipation learning paradigm. On every trial, the name and portrait of one suspect was presented along with four groups of buttons that denoted the cue values of each of the four cues. The learning order of cues matched the validity hierarchy of the respective condition. For every cue, participants had to guess (first trial) or recall (further trials) the correct cue value. Afterward, they received feedback by showing the correct cue value verbally (verbal conditions) or by adding the cue value to a full-length portrait (pictorial conditions; see Fig. 3). Every suspect was repeated until cue values were recalled without error. Afterward, all ten suspects were presented consecutively, regardless of any errors in recall. Learning was continued until 90 % of cue values were recalled correctly. The order of suspects, as well as the order of cue values within each cue, was randomly determined.

After the learning phase, a cue hierarchy was established. Participants were told that eye witnesses had seen the alleged perpetrator and could remember her clothes (leather jacket, polo shirt, leggings, and tote bag). As in the original studies by Bröder and Schiffer (2003, 2006), cue validity was manipulated via the number of witnesses who agreed on a certain clothing attribute: The more witnesses agreed, the higher the probability that a suspect who had worn this clothing was the murderer.^3^ Information about validities was presented verbally before, but not during, the subsequent decision phase.

On each trial of the decision phase, participants were presented portraits and names of two suspects (Fig. 3). They had to decide which suspect was more likely to have committed the murder by using the acquired knowledge about cue values and the cue hierarchy. This information had to be retrieved from memory. Fifty-three decisions had to be made, consisting of a full paired comparison of 10 patterns, with 8 patterns being presented twice.^4^ The order of paired comparisons, as well as the position of the portraits, was determined at random, with the only restriction being that identical paired comparisons were not presented consecutively.

**Fig. 4:**
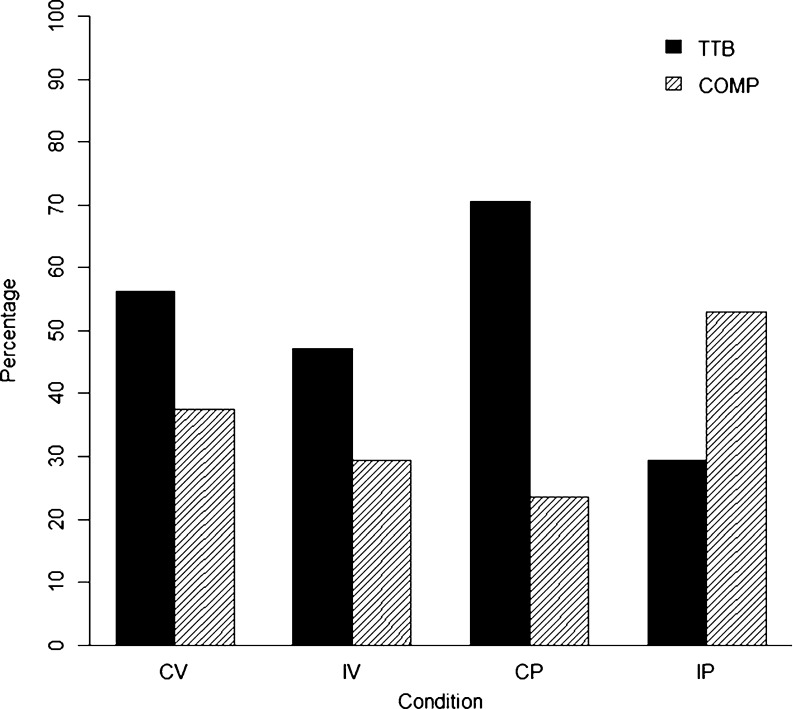
Percentage of participants using TTB or a compensatory strategy (COMP: WADD, EQW) in the congruent verbal (CV), incongruent verbal (IV), congruent pictorial (CP), and incongruent pictorial (IP) conditions

### Results

#### Classification of decision strategies

An outcome-based maximum-likelihood method was used to identify individual decision strategies. The method is described in detail in Bröder (2010). In a nutshell, the method estimates the likelihood of the data, given the strategies TTB, WADD, EQW, and response errors *ε* that differ between individuals but are assumed to be constant across trials. Each participant was classified according to the highest likelihood, if the estimated response error $\hat{\varepsilon }$ for the best-fitting model was less than .40 (Bröder & Schiffer, 2003). Otherwise, the pattern was classified as a random-guessing strategy. If two strategies exhibited identical likelihoods, the response pattern remained unclassified.

Table 2 reports the strategy classification frequencies across conditions. Frequency distributions of TTB and a pooled compensatory decision strategy (COMP: WADD and EQW) were compared across experimental conditions via follow-up chi-squared tests (Agresti, 2002). To test whether the application of a certain decision strategy depends on cue salience instead of cue format, the following partitioning was carried out. In a first step, the two verbal conditions were tested against each other, revealing no significant influence of stimulus material on strategy application, *G* ^2^(1, *N* = 28) = .007, *p* = .93, $\hat{w}$ = .02. Hence, the different semantic content of cues did not influence strategy application. In the next step, aggregated data for both verbal conditions was tested against the CP condition. Again, no difference in the frequency distribution of strategies was found, *G* ^2^(1, *N* = 44) = .95, *p* = .33, $\hat{w}$= .15. However, comparing frequency distributions of the IP condition against the other three conditions revealed significant differences in strategy application, *G* ^2^(1, *N* = 58) = 3.96, *p* = .046, $\hat{w}$ = .26. A preponderance of compensatory decision making was found only in an IP condition, in which cue validity and salience were negatively correlated (Fig. 4).

**Table 2:** Strategy classification frequencies depending on condition

Condition		Strategy classification				
		TTB	WADD	EQW	Guess	Unclass
Verbal	Congruent	9	1	5	1	0
		56 %	6 %	31 %	6 %	0 %
	Incongruent	8	3	2	3	1
		47 %	18 %	12 %	18 %	6 %
Pictorial	Congruent	12	3	1	0	1
		71 %	18 %	6 %	0 %	6 %
	Incongruent	5	3	6	2	1
		29 %	18 %	35 %	12 %	6 %

*Note*. TTB, take the best; WADD, weighted additive rule; EQW, equal weight rule; Guess, guessing (percentage of predicted inferences, < 60 %); Unclass, unclassified pattern (identical likelihoods for two strategies)

#### Decision time data

**Fig. 5:**
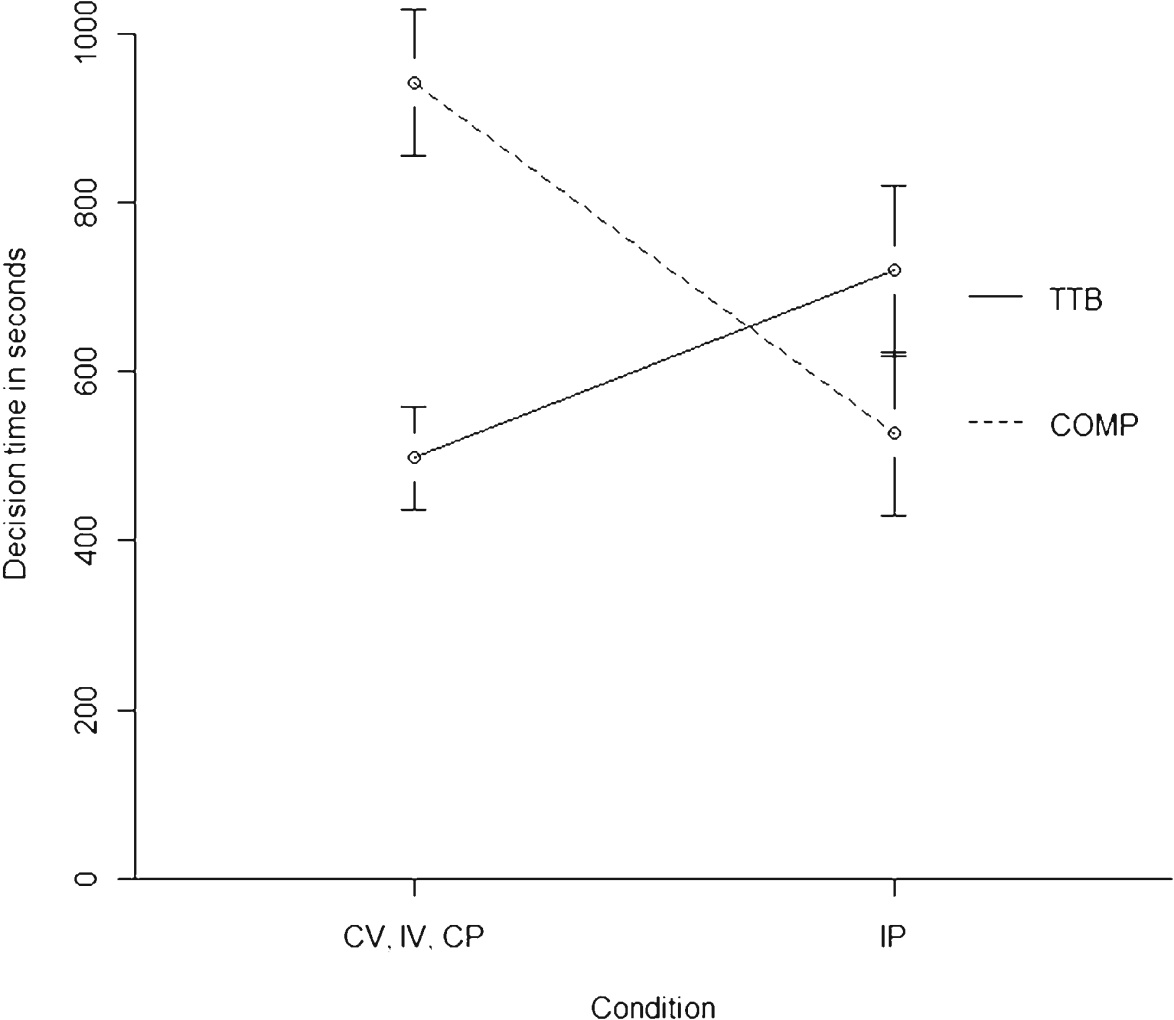
Decision times (and standard errors) for different strategies and conditions. TTB, take the best; COMP, compensatory strategies; CV, congruent verbal condition IV, incongruent verbal condition; CP, congruent pictorial condition; IP, incongruent pictorial condition

First, the overall time to complete the decision phase was log-transformed for each participant to reduce skewness. Log-transformed decision times were then regressed on decision strategy, experimental condition, and the interaction of the two.^5^ Decision strategy significantly predicted decision time, *B* = −.26, *SE*(*B*) = .08, *t*(50) = −3.40, *p* = .001. As was expected, TTB users were faster in making decisions than were COMP users, averaged across experimental conditions. More important, decision strategy interacted with experimental condition such that TTB users were slower than COMP users in the IP condition, whereas the opposite was true in the remaining three conditions (Fig. 5). In line with that interpretation, the contrast1 × strategy interaction effect was significant, *B* = .15, *SE*(*B*) = .04, *t*(50) = 3.33, *p* = .002, whereas the remaining contrast × strategy interaction effects were not significant (*p* > .22). The interaction between decision strategy and experimental condition explained 18 % of the variance in the decision times.

## Discussion

The results of this study are clear-cut: There was no format effect in the CP condition, whereas it was present in the IP condition. In the CP condition, participants apparently used TTB in the same manner as with a verbal cue format. Hence, there is no reason to believe that the pictorial format per se led to a facilitated retrieval of cue information, as Bröder and Schiffer (2003, 2006) suggested. If, however, the least valid cues were the most salient ones, more participants used compensatory decision strategies. This suggests that salient cues were retrieved with high probability regardless of their validity and were then integrated into judgments.

Manipulating the cue validity hierarchy via the number of witnesses who agree on a certain cue might be seen as a weakness in the cover story. Participants might not have believed that less salient features were agreed upon by more witnesses in the IP condition. We cannot strictly rule out this possibility with the present data, but we recently replicated the effect with another cover story for which this alternative interpretation was implausible (Platzer, Bröder, & Heck, 2012). Participants had to rate the probability of a painting being an art forgery. The motif, the shape of the background, the signature, and the kind of picture frame served as cues. Salience hierarchy was determined in a pilot study, indicating that motif was the most salient cue, followed by background, signature, and picture frame. Validity was manipulated via the number of experts who agreed that a certain feature argued for a forgery. In contrast to the present study, there is no reason to believe in a positive correlation between salience and validity in this new setting.

To rule out the possibility that a simple noncompensatory strategy based on cue salience can better account for the data than can a compensatory strategy, we also fitted a simple decision heuristic that we refer to as “take the most salient” (TTS). TTS searches cues sequentially in the order of cue salience and chooses the option with the critical cue value on the first discriminating cue. Since TTS's predictions are identical to those of TTB in the CP condition, TTS was fitted only in the IP condition. No individual in the IP condition was classified as a TTS user.

Our results have several theoretical and practical implications. First, they indirectly corroborate Gigerenzer and Todd's (1999) hypothesis that noncompensatory strategies are used in memory-based decisions because of costly information retrieval. If cues are visually salient, however, they are retrieved with greater ease. Compensatory strategies can be used, since less valid cues also come to mind readily. Noncompensatory decision making is even impaired, because automatically retrieved invalid cues have to be ignored. This was corroborated by reaction time data. Whereas TTB showed shorter reaction times in both the verbal and the CP conditions, the opposite was true for the IP condition. This can be interpreted such that ignoring less valid but salient cues is costly in the sense of cognitive effort needed. Additionally, compensatory decision making was faster in the IP condition, as compared with all other conditions, indicating that less valid but salient cues were retrieved more easily. In sum, reaction time analyses validate the salience effect. Salient cues can be retrieved easily. Integrating them into a judgment is even easier than ignoring them.

These results are in line with a finding from Bröder and Gaissmaier (2007). The authors disentangled cue validity and learning order, which either matched or not. There were some participants who apparently retrieved cues in the order they had learned them, rather than in the order of validities. Consistent with the results reported in this article, it seemed to be difficult for participants to ignore irrelevant information if it came to mind readily.

Summing up, this experiment shows that the interaction between validity and salience probably determined strategy selection in the experiments reported by Bröder and Schiffer (2003, 2006). Our results, as well as research within the field of inferences from givens (Newell & Lee, 2010), did not provide any evidence for a main effect of cue presentation format. However, we cannot rule out the existence of the format effect in general. In order to reject the format hypothesis in memory-based decisions, a comparison of verbal and pictorial stimuli without any salience differences would be necessary, which is (arguably) hard to establish.

An interesting avenue for further research is the question of whether the salience effect is involuntary (cues cannot be ignored) or, rather, strategic, reflecting an (erroneous) misattribution of retrieval fluency on validity (e.g., Unkelbach, 2006).

Practically, the result corroborates the common wisdom of marketing strategies that aim at highlighting positive aspects of a product, combined with salient images of those positive aspects. These may indeed come to mind most quickly and influence decisions regardless of their validity for assessing product quality.

## Electronic supplementary material

ESM 1 (PDF 91 kb)

## Footnotes

1 We thank one of the reviewers for the advice to test this confound empirically. Corresponding pilot studies are reported in the supplementary materials.

2 Imagine a suspect with cue pattern 1–1–0–0, with 1 denoting a critical cue value and 0 denoting a noncritical cue value. Since the critical cue values were leather jacket, polo shirt, leggings, and tote bag, a suspect in the CP condition wore a leather jacket and a polo shirt and two noncritical pieces of clothing. However, a suspect in the IP condition with an inverted validity hierarchy wore a tote bag and leggings and a noncritical shirt and leather jacket.

3 The term *cue validity* is used in a broader sense here than, for example, in PMM theory (Gigerenzer, Hoffrage, & Kleinbölting, 1991), where it is measured as a conditional probability of a criterion value, given a cue value. In the broader Brunswikian sense, the validity of a cue is its statistical relation to a judgment criterion. This can be measured in many different ways—for example, with correlations or beta weights (e.g., Martignon & Hoffrage, 2002). Our instructions made clear how each cue was related to the criterion, and a cue validity hierarchy was established by the cover story. Hence, cue validities were not learned by observing the frequencies of co-occurrences in an environment (as assumed by PMM), but they were provided to participants via instruction. Many empirical tests of fast and frugal heuristics have used this method, and since the experiment reported here was a direct replication of those in Bröder and Schiffer (2003, 2006), we employed their methodology for comparability. Testing PMM theory was not the aim of our experiment, but adding a feedback training phase for cue learning might be another experimental option if a test of PMM theory is intended.

4 Only 8 out of 45 paired comparisons discriminated between TTB and compensatory strategies (WADD, EQW). Hence, these eight trials were presented twice to allow for a more reliable strategy classification.

5 Decision strategy was contrast-coded with TTB = +1 and COMP = −1. Experimental conditions were transformed into three Helmert-type contrasts, with contrast1 comparing the IP condition with the other three conditions (IP = +3, others = −1 each), contrast2 comparing CP with the remaining two (IP = 0, CP = +2, IV and C = −1), and contrast3 comparing IV with CV (IP and CP = 0, IV = +1, CV = −1). Thus, the regression equation consisted of seven predictor terms, including interaction effects between decision strategy and contrasts.
