# Supplementary material for: Most people do not ignore salient invalid cues in memory-based decisions
Source: Psychon Bull Rev. 2012 May 4;19(4):654–61. doi: 10.3758/s13423-012-0248-4 (PMC3394236; doi:10.3758/s13423-012-0248-4)
Supplement: Supplementary file 1 — (PDF 91 kb) [file 13423_2012_248_MOESM1_ESM.pdf]

Online Supplementary Materials

Pilot studies: Measuring cue salience

The aim of the pilot studies was to determine the visual salience of the pictorial cues used by Bröder and Schiffer (2003, 2006) and in the main experiment reported in this article.

Pilot Study 1: Confounding in the original work

In the original studies, Bröder and Schiffer (2003, 2006) did not control for visual salience of their pictorial cues. If less valid cues had been particularly salient, compensatory decision making might have been facilitated, since salient cues come to mind more easily. To substantiate the assumption that validity and visual salience were confounded in the original studies, visual salience of the original materials was tested empirically in the present research.

Method

Participants. 16 participants from different fields of study and different occupations volunteered in Pilot Study 1 (12 female, mean age 23.50, *SD*=4.00). The experimental sessions took 6.5 minutes on average. Participants received 1.50 Euros.

Materials and design. The cover story used by Bröder and Schiffer (2003, 2006) was about a criminal case that could be solved by identifying the perpetrator. The cues concerned attributes of the suspects. A total of 10 female suspects were presented, who differed with regard to four cues: Accompanying dog's race (Dalmatian, Spaniel, Dachshund), coat (cardigan, blazer, jeans), trousers (leather, linen, jeans), and shirt color (yellow, green, red). The aim of the first pilot study was to measure these four cues' visual salience.

Procedure. Visual salience was determined by analyzing similarity ratings between pairs of suspects. In each trial two suspects were presented simultaneously on screen, and participants had to judge overall similarity on a 7-point scale (1="not similar at all" to 7="very similar"). Participants completed 90 trials altogether with 45 comparisons being presented twice. The two

suspects in each trial differed with regard to either 1, 2, 3, or 4 cues. Visual salience was operationalized as the extent to which the mismatch on a certain cue (suspects exhibit different cue values) affected similarity ratings.[1]

Results

Salience hierarchy. To establish a salience hierarchy of cues, similarity ratings were regressed on the four dichotomous predictors: For each cue (dog's race, coat, trousers, shirt color) a mismatch (i.e., different cue values between suspects in a trial) was coded as 0, and a match (i.e., same cue values between subjects) was coded as 1. For example, a trial with a value of 0 on all four predictor variables indicates that the two suspects in this trial differed with regard to their dog, their coat, their trousers, and their shirt color. Since trials were nested within participants, hierarchical linear modeling was used to estimate regression parameters. A random coefficient model with random intercepts and random slopes was used; the correlation between random coefficients was restricted to Zero. The results indicated that the most valid cue (*dog*, B=1.02, $t(1,16)$=5.65, p<.001) was less salient than the second most valid cue (*coat*, B=1.56, $t(1,16)$=7.28, p<.001), followed by the two least valid cues (*trousers*, B=0.86, $t(1,16)$=7.06, p<.001; *shirt color*, B=0.45, $t(1,16)$=4.12, p=.001).

Discussion

The results of Pilot Study 1 show evidence for a confounding in the original studies. Since the most valid cue was less salient than the second most valid cue, this might have influenced strategy selection.

Pilot Study 2: Establishing an unambiguous salience hierarchy

The aim of Pilot Study 2 was to develop pictorial stimuli that exhibit an unambiguous salience hierarchy. This was necessary because we wanted to manipulate the congruency between cue validity and cue salience in the main experiment. Since the virtual dress room used by Bröder

and Schiffer (2003, 2006) to create their stimulus materials was no longer available, we had to use different stimuli. However, we tried to create stimulus materials that are as close as possible to the original ones.

Method

Participants. 50 participants from different fields of study and different occupations volunteered in Pilot Study 2 (42 female, mean age 23.12, *SD*=4.31). The experimental sessions took 6.6 minutes on average. Participants received either course credit or 1.50 Euros.

Materials and design. Based on the cover story of an invented criminal case that could be solved by identifying the perpetrator, the visual salience of four different cues had to be determined. Again, the cues concerned attributes of the suspects. The female suspects differed with regard to the articles of clothing they had worn at the time the actual perpetrator left the site of crime. Different types of coats (leather jacket, cardigan, blazer), tops (polo shirt, shirt, blouse), trousers (jeans, leggings, linen trousers), and bags (tote bag, hand bag, wrist purse) served as cues. Stimuli were selected carefully to ensure that cue values within each cue did not differ in salience (e.g., by dispensing with bright colors and keeping size constant).

A female dummy was created, exhibiting all possible combinations of cue values, resulting in 81 pictures (3^4 combinations). The female dummy and the stimulus combinations were created by means of a WWW virtual dress room (http://www.mvm.com).

Procedure. As in Pilot Study 1, participants had to judge overall similarity of two simultaneously presented female dummies on a 7-point scale (Figure 1). The study consisted of 120 paired comparisons that were randomly selected for each participant. Simultaneously presented dummies differed on one up to four cues and the visual salience was operationalized as the extent to which the mismatch on a certain cue (dummies exhibit different cue values) affected similarity ratings.

Results

Salience hierarchy. As in Pilot Study 1 hierarchical linear modeling was used to estimate regression parameters. The results indicated a distinct salience hierarchy with *coat* being the most salient cue (B=1.64, $t(1,49.89)$=15.17, p<.001), followed by *top* (B=.69, $t(1,50.05)$=10.34, p<.001), *trousers* (B=.66, $t(1,49.86)$=10.54, p<.001), and *bag* being the least salient cue (B=.20, $t(1,49.70)$=4.51, p<.001).

Discussion

Pilot Study 2 aimed to determine the visual salience of the four cues used later on in the experiment. Stimulus selection was based on two principles: First, cues should differ in visual salience to ensure an unambiguous salience hierarchy. The results indicate the following order of salience from the most to the least salient cue: coat, top, trousers, and bag. Second, cue values within each cue should be similar in salience to ensure a stable salience hierarchy on a cue level. Besides selecting stimuli carefully, multiple regression analyses were run, in which all trials with a specific cue value were excluded each time. If all cue values within each cue are equally salient, this should not change salience hierarchy on a cue level. Results indicated almost no change in salience hierarchy.
